# Hooded seal (*Cystophora cristata)* pups ingest snow and seawater during their post-weaning fast

**DOI:** 10.1007/s00360-016-1048-3

**Published:** 2016-11-09

**Authors:** Pauke C. Schots, Marie E. Bue, Erling S. Nordøy

**Affiliations:** Department of Arctic and Marine Biology, UiT-The Arctic University of Norway, Breivika, 9037 Tromsø, Norway

**Keywords:** Tritiated water, Mariposia, Water balance, Hooded seal, Post-weaning fast, Homeostasis

## Abstract

The purpose of this study was to evaluate the importance of exogenous water intake (snow/seawater) in hooded seal (*Cystophora cristata*) pups during their post-weaning fast. In this study, five hooded seal pups had ad lib access to snow and seawater for the first 12 and last 21 days of their post-weaning fast, respectively. Total body water and water flux were determined during both exposure periods by use of the tritiated water method. Blood samples were collected to monitor changes in hematocrit, plasma urea and plasma osmolality. Body mass loss was on average 0.36 kg day^−1^. Average total body water changed from 15.7 to 11.4 L, while total water influx changed from 15 to 18 mL day^−1^ kg^−1^ during snow and seawater exposure, respectively. Of this influx an average of 35% can be attributed to metabolic water, while approximately 8% was due to respiratory water influx. Interestingly, 56 and 58% of the total water influx was due to snow and seawater ingestion, respectively, amounting to 8 mL day^−1^ kg^−1^ snow (counted as liquid water) and 10 mL day^−1^ kg^−1^ seawater. Based on the results of the plasma parameters it is concluded that fasting hooded seal pups maintain water balance and homeostasis when access to snow or seawater is permitted. It is further concluded that snow and seawater intake, in addition to metabolic and respiratory water, is important for maintenance of water balance and excretion of urea during the post-weaning fast of hooded seal pups.

**Electronic supplementary material:**

The online version of this article (doi:10.1007/s00360-016-1048-3) contains supplementary material, which is available to authorized users.

## Introduction

The hooded seal (*Cystophora cristata,* Erxleben, 1777) lives in the North Atlantic where it breeds in four different geographical areas: the Davis Strait, off the coasts of Labrador and Newfoundland, the Gulf of St Lawrence, and the “West-Ice” being the pack ice between Jan Mayen and Greenland (Bowen et al. 1985; Kovacs and Lavigne 1986; Lydersen et al. 1997). The pups from the latter group are born in the second half of March (Rasmussen 1960). As a consequence of the unstable and unpredictable environment wherein pups are born, adaptations have evolved to survive. These adaptations consist of a large size at birth, prenatal blubber deposits (Oftedal et al. 1993), prenatal moult (Oftedal et al. 1991), short lactation period, and efficient postnatal fat and energy transfer from milk to body tissue (Oftedal et al. 1993). Hooded seals weigh around 20–24 kg at birth. The lactation period is short but intense, lasting only 4 days. During this period they gain 7 kg per day, resulting in a body mass of more than 42 kg at weaning (Bowen et al. 1987, 1985; Kovacs and Lavigne 1992; Lydersen et al. 1997). After the lactation period, the hooded seal pups go through a post-weaning fast. The fast lasts for about 1 month (Bowen et al. 1987; Folkow et al. 2010) in which they loose on average 0.4 kg day^−1^ (Bowen et al. 1987). During their fast, the hooded seal pups of the “West-Ice” population passively follow the pack ice in a south-western direction. During this period the pups remain in the vicinity of the pack ice where they have access to fresh water in the form of snow and ice. From the moment they leave the pack ice, however, they are pelagic in open seawater for 2.5 months before returning to the ice (Folkow et al. 2010).

To survive in a hyperosmotic environment, physiological adaptations are required to conserve water and avoid dehydration. In pinnipeds, water can leave the body through three different routes: respiratory evaporation, cutaneous evaporation and urine production. Although hooded seals have sweat glands (Kovacs and Lavigne 1986), pinnipeds do not appear to sweat (Ridgway 1972; Whittow et al. 1972). One of the water conserving adaptations found in pinnipeds is nasal counter current heat exchange. Through this morphological adaptation the expired air temperature is lowered, which reduces the respiratory water loss (Folkow and Blix 1987; Huntley et al. 1984). Another morphological characteristic are the reniculate kidneys, found in cetaceans and pinnipeds (Ortiz 2001). Reniculate kidneys have an increased medullary thickness allowing the production of urine with an increased osmolality to reduce urinary water loss (Bester 1975; Vardy and Bryden 1981). Marine mammals are able to produce urine with an osmolality well above that of seawater (1000 mOsm kg^−1^) (Ortiz et al. 1996; Skog and Folkow 1994; Storeheier and Nordøy 2001). The highest urine osmolality measured in a marine mammal was 2658 mOsm kg^−1^ in a common bottlenose dolphin (*Tursiops truncatus*) (Ridgway 1972). The highest osmolality registered in seals was that of the Baikal seal (*Pusa sibirica*), measuring 2374 mOsm kg^−1^ (Hong et al. 1982).

During fasting, water conservation mechanisms need to be even more efficient since there is no free water entering the animal with food. One of the mechanisms found in fasting northern elephant seal (*Mirounga angustirostris*) pups is a reduction in glomerular filtration rate, which reduces the urinary water loss (Adams and Costa 1993; Pernia et al. 1980). Another characteristic of the fast is that protein catabolism decreases (Adams and Costa 1993), reducing the nitrogen load on the kidneys. The reduced nitrogen load, together with an increased urine osmolality, also decreases urinary water loss.

A urine osmolality above that of seawater is a prerequisite for a net water gain from mariposia (voluntary drinking of seawater). Despite that seals are capable of producing urine with an osmolality above that of seawater Irving et al. (1935) concluded that young captive harbour seals (*Phoca vitulina*) did not drink seawater, but derive enough water from the food to maintain their water balance. Albrecht (1950) observed that harbour seals do not tolerate seawater and that they vomit and get diarrhoea after orally administering a seawater volume of 3.3% of their body mass. Storeheier and Nordøy (2001), on the other hand, observed that after a seawater bolus administration (2% of the body mass) through a stomach tube in harp seals (*Phoca groenlandica*), urine osmolality remained stable and above seawater levels. They further observed that the animals were able to concentrate urinary sodium and chloride to levels above seawater. Additional support for the tolerance of seawater was found by Depocas et al. (1971). They found that harbour seals do in fact ingest seawater. However, since this volume was small, it was concluded that this was an accidental ingestion due to the intake of food under water and not due to deliberate mariposia. They also concluded that fasting harbour seals derive enough oxidative water to maintain their water balance. These conclusions were the general assumptions in later studies on water flux, water balance and food intake in marine mammals (Folkow and Blix 1987; Hong et al. 1982).

However, other studies on several marine mammal species have shown that mariposia does occur (Costa 2009; Suzuki and Ortiz 2015). It has been observed that Australian fur seals *(Arctocephalus forsteri)*, Steller sea lions *(Eumetopias jubatus)*, Northern fur seals *(Callorhinus ursinus)* and California sea lions *(Zalophus californianus)* drink both from tidal pools and from the sea (Gentry 1981). Skalstad and Nordøy (2000) showed that mariposia accounts for 14 and 27% of the water influx in young fed hooded seals and harp seals, respectively. Common dolphins (*Delphinus delphis)* drink up to 0.8 L day^−1^ (Hui 1981), whereas pilot whales (*Globicephala scammoni)* can drink up to 1.8 L day^−1^ (Tefler et al. 1970). Sea otters (*Enhydra lutris*), feeding on clams, derive 33.8% of the total water influx from the ingestion of seawater (Costa 1982), and Bentley (1963) concluded, based on the urine concentration, that fasting humpback whales (*Megaptera novaeangliae)* swallow seawater, but without a net water gain.

For an animal to obtain a net gain of water from mariposia, it should not only be able to concentrate urine above the concentration of seawater. The concentration of Na^+^ and Cl^−^ in urine should also be higher than in seawater (Albrecht 1950; Tarasoff and Toews, 1972). Although the latter is usually not the case, How and Nordøy (2007) observed that dehydrated harp seals can concentrate Na^+^ and Cl^−^ in their urine to 540 and 620 mM, respectively, which is above the concentrations found in seawater (444 mM Na^+^ and 535 mM Cl^−^). Thus, harp seals were able to restore water balance through mariposia. Other suggested reasons for mariposia are facilitating thermal regulation in fasting animals that inhabit warm environments (Gentry 1981), increasing the urinary osmotic space to excrete urea (Costa 1982; Wolf et al. 1959) and maintaining mineral balance (Ridgway 1972; Ridgway and Venn-Watson 2010).

In this study, five hooded seal pups were exposed to two types of exogenous water sources, snow and seawater, during their post-weaning fast. With the tritiated water method, total body water and water influx rates were calculated. From these rates the water influx through snow and seawater was calculated. The aims of this study were (1) to determine if, and to what extent, hooded seal pups ingest snow and seawater during their post-weaning fast, and (2) to determine the effect of snow and seawater ingestion on water balance. It is hypothesized that the hooded seal pups studied here do ingest snow and seawater, and that the intake of exogenous water is necessary to maintain water balance during fasting.

## Materials and methods

### Animals

Five recently weaned hooded seal pups were captured on the pack ice of the Greenland Sea in late March 2014. The animals were kept on board RV “Helmer Hanssen” and brought to the approved animal research facility of the Department of Arctic and Marine Biology at UiT-The Arctic University of Norway. On board of RV “Helmer Hanssen” the ambient air temperature was around 0 °C and the animals had ad lib access to snow that was provided on a daily basis. Upon arrival at the research facilities, 12 days after capture, the animals were introduced to a 42,000-L seawater pool under continuous water flow until the end of the experiment, 33 days after capture. While four of the five animals seemed healthy throughout the experiment, one of the animals died on the 28th day of the experiment for unknown reasons. The temperature at the research facility was kept around 6 °C and the day length was simulated as at 70°N. The animals went through a period of post-weaning fast and were therefore not fed. All experiments were conducted in accordance with the Norwegian Animal Welfare Act and were approved by the National Animal Research Authority of Norway (approval #6216).

### Sample collection

The ingestion of snow and seawater, as well as other water influx rates, total body water and body composition were measured with the tritiated water method. Blood samples were repeatedly collected to measure changes in plasma parameters. Prior to tritiated water injections and blood sampling, the animals were loosely restrained on a specially designed board. A dose of 0.25 mL tiletamine-zolazepam (50 mg mL^−1^ tiletamine, 50 mg mL^−1^ zolazepam, Zoletil Forte Vet, Virbac Laboratories, France) was administered intramuscularly to anaesthetize the animal. An incubation time of at least 10 min was permitted for the anaesthesia to immobilize the animal before further handling.

The tritium isotope (Perkin Elmer, Boston, USA) was diluted in physiological saline (0.9% (w.v) sodium chloride, B. Braun Melsungen AG, Melsungen, Germany). 8–9 mL tritiated water (13 µCi mL^−1-^) was injected on the day of capture into the intravertebral extradural vein through a 16-cm long catheter (Selacon-T™ 16G/1.70 × 160 mm, The Hague, The Netherlands) at the level of the fourth lumbar vertebra. Prior to the injection of tritiated water a blood sample was collected to measure the plasma’s background level of radioactivity. After an equilibration period of 60 and 90 min two other blood samples were collected to calculate total body water. Additional blood samples were collected 3, 5, 12, 19, 26 and 33 days after capture. Tritiated water was reinjected (8–9 mL, 50 µCi mL^−1^) 12 days after capture, to calculate changes in total body water, body composition, and water influx during snow exposure. A third and final tritiated water injection (8–9 mL, 50 µCi mL^−1-^) was given 33 days after capture to calculate changes in total body water, body composition and water influx during seawater exposure.

After each blood sampling the animals were weighed with a DHS crane weight (Scaleit, Norway), with a precision of ±0.15%. All blood samples were collected in 10.0 mL vacutainers (BD, LH 170 I.U., Plymouth, UK). The samples were stored on ice and centrifuged for 15 min at 2500-rpm within 2 h after sampling. The plasma was separated from the precipitate and transferred to 2 mL cryovials (VWR, Leuven, Belgium). All plasma samples were frozen in liquid nitrogen and stored at −80 °C until further analyses. About half of the plasma of each sampling moment was used to measure osmolality and urea concentrations. The other half of the plasma was deproteinized using 70% perchloric acid (HClO_4_ ACS reagent 70%, Sigma–Aldrich, St. Louis, USA). The tritium concentration of the deproteinized plasma sample was determined by standard liquid scintillation techniques using a beta liquid scintillation counter (1900 TR Packard, A Canberra Company, Oslo, Norway). The plasma samples were deproteinized to avoid quenching.

### $r_{{{\mathbf{H}}_{2} {\mathbf{O}}}}$ and *r*_W_ calculations and corrections

The concentration of the tritium isotope measured by the scintillation counter was corrected for the water content of the plasma prior to further calculations. Total body water was calculated following Eq. 1 where N is total body water in mL, i.d. the injected dose of the tritium isotope and S.A. the specific activity of the isotope in the body water.

1 $$N = \frac{{{\text{i}} . {\text{d}} .}}{{{\text{S}} . {\text{A}} .}}.$$

**Fig. 1:**
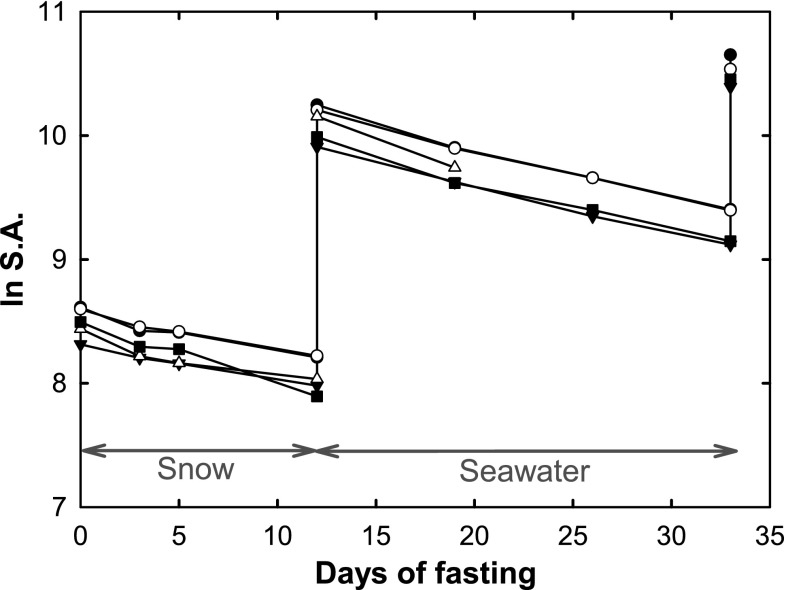
Natural logarithm of the plasma specific activity over time, indicating the fractional turnover rate of tritiated water (*K*_2H_). Three different tritium injections on the day of capture and 12 and 33 days after capture in five fasting hooded seal pups which coincide with an elevated specific activity. *Arrow lines* indicate the time periods of snow and seawater exposure

The natural logarithm of the S.A. of tritium in the plasma was plotted against time (Fig. 1). The inclination of the decay (–K_2H*_) of the S.A. in the plasma is the fractional turnover rate of the isotope (Eq. 2). $H_{1}^{*}$ and $H_{2}^{*}$ are the initial and final specific activities of the tritium isotope in the body water and t is the elapsed time (Nagy and Costa 1980).

2 $$K_{2H*} = \frac{{{ \ln }(H_{1}^{*} /H_{2}^{*} )}}{\Delta t}.$$

The daily change in body water (*K*_*N*_) was calculated with Eq. 3 where *N*_0_ and *N*_1_ is the initial and final total body water in mL, respectively.

3 $$K_{N} = \frac{{\ln N_{0} - \ln N_{1} }}{\Delta t}.$$

It was assumed that total body water decreased exponentially over time since the animals’ body mass decreased exponentially, due to their fast (Ortiz et al. 1978). Equation 16 from Lifson and McClintock (1966) was used to calculate the average daily water efflux rate ($r_{{{\text{H}}_{ 2} {\text{O}}}}$) in mL day^−1^ (Eq. 4). The final estimates for ${\text{r}}_{{{\text{H}}_{2} {\text{O}}}}$ were corrected for exchange and fractionation (Lifson and McClintock 1966; Nagy and Costa 1980).

4 $$r_{{{\text{H}}_{ 2} {\text{O}}}} = \frac{{N_{0} K_{N} (K_{2H*} - K_{N} )\Delta t}}{{1 - \exp ( - K_{N} \Delta t)}}.$$

The average daily water influx rate (*r*_w_) in mL day^−1^ was calculated with Eq. 5, according to Lifson and McClintock (1966) and Ortiz et al. (1978). The total water influx rate can be divided in respiratory water influx (H_2_O_respiratory_), metabolic water (H_2_O_metabolic_) and the ingestion of snow or seawater (H_2_O_snow/seawater_).

5 $$r_{\text{w}} = |r_{{{\text{H}}_{ 2} {\text{O}}}} | - \frac{\Delta N}{\Delta t}.$$

### H_2_O_respiratory_

The respiratory water influx rates (in mL day^−1^) were calculated with Eq. 6, according to Folkow and Blix (1987), with a known ambient air temperature and assuming a 100% relative humidity of the air. M_W_ is the mass of evaporated water in mg s^−1^ kg^−0.75^, *P*_W_ the water vapour pressure in the inhaled air in mmHg, *V*_L_ the respiratory minute volume in L min^−1^ kg^−0.75^, *M* the molar mass of water, *T*_g_ the gas temperature in °K, *R* the gas constant (62.63 mmHg L °K^−1^ mol^−1^) and *t* the time constant (60 s min^−1^).

6 $$M_{\text{W}} = \frac{{P_{\text{W}} V_{\text{L}} M}}{{T_{\text{g}} R t}}.$$

The respiratory minute volume (*V*_L_) was calculated following Eq. 7 (Folkow and Blix 1987). Where MR is the metabolic rate based on the caloric equivalent of fat and protein loss. Protein and fat loss are obtained using Eqs. 8 and 9.

7 $$V_{\text{L}} = 0.042 \cdot {\text{MR}} + 0.119.$$

### H_2_O_metabolic_

Metabolic water (in mL day^−1^) was calculated as the water derived from fat and protein oxidation. Total body fat (TBF) and total body protein (TBP), as percentages of body mass, were calculated following Eqs. 8 and 9, respectively (Reilly and Fedak 1990). %N is total body water as percentage of total body mass. The metabolic water was subsequently calculated using Eq. 10

8 $$\% {\text{TBF}} = 105.1 - 1.47 \cdot\% N,$$

9 $$\% {\text{TBP}} = 0. 4 2 \cdot \% N - 4. 7 5 ,$$

10 $$\begin{aligned} {\text{H}}_{2} {\text{O}}_{{{\text{metabolic }}}} & = 1.071\cdot\left( {{\text{TBF}}_{1} - {\text{TBF}}_{2} } \right) \\ & \quad + 0.396\cdot\left( {{\text{TBP}}_{1} - {\text{TBP}}_{2} } \right). \\ \end{aligned}$$

### H_2_O_snow/seawater_

The water influx rate through the ingestion of snow or seawater (in mL day^−1^) was calculated as the difference between the total daily water influx rate and the metabolic and respiratory water influx rates:

11 $${\text{H}}_{ 2} {\text{O}}_{\text{snow/seawater}} = r_{\text{W}} - ({\text{H}}_{ 2} {\text{O}}_{\text{respiratory}} {\text{ + H}}_{ 2} {\text{O}}_{\text{metabolic}} ).$$

### Blood samples

Right after blood sampling an aliquot of blood was used to determine the haematocrit percentage. A capillary vial (Aris, Soda Lime glass 80iu mL^−1^, Am-Heparinized Vitrex, Herlev, Denmark) was filled with blood and centrifuged for 10 min at 3000 rpm using a microcentrifuge (Hettich-Zentrifugen, Tuttlingen, Germany). The haematocrit percentage was read on the haematocrit scale.

Plasma samples were analysed for urea concentrations and the osmolality. The plasma samples were thawed up over night. Urea concentrations were measured with a Reflotron system (Reflotron, Mannheim Boehringer, Mannheim Germany). The osmolality was determined by freezing-point osmometry (Osmomat030, Gonotec, Berlin, Germany).

### Statistics

Total body water, water influx rates and blood parameters obtained throughout the experiment were compared using a paired Student’s *t* test using SPSS statistics (IBM version 22). Values are given as averages ± standard deviation.

## Results

**Fig. 2:**
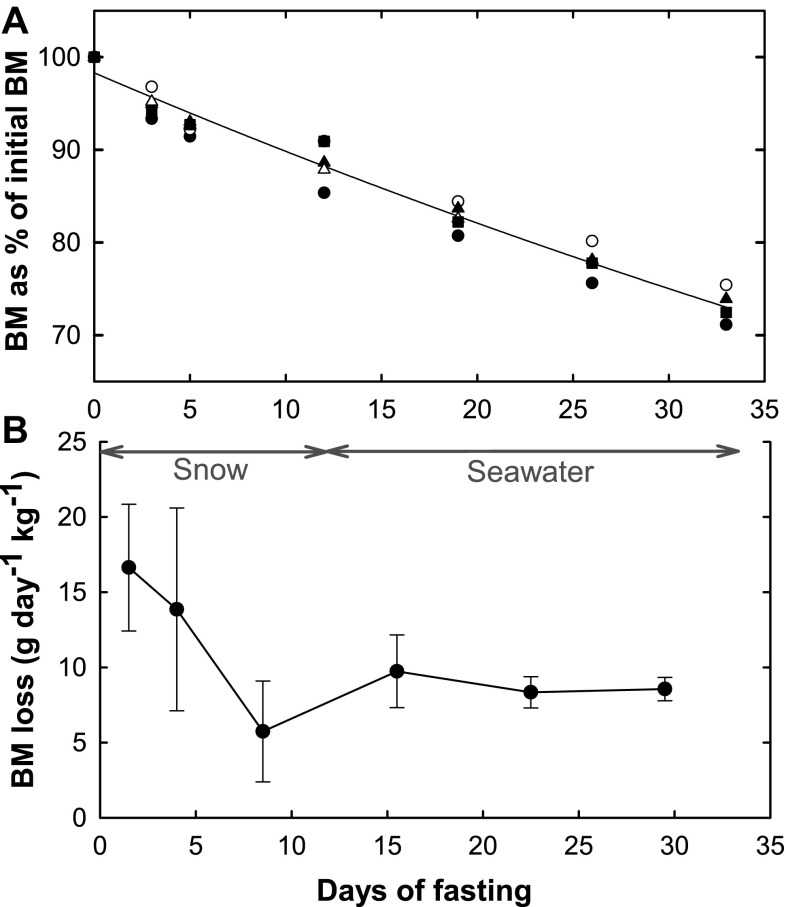
**a** Body mass loss as a percentage of initial body mass of five hooded seals pups during a 33 days long post-weaning fast. The average decrease in body mass of all five seals can be described by the function BM = 98.309 e^−0.009*t*^ (*R* ^2^ = 0.98) where BM is body mass in percentage of initial body mass and t is time in days of fasting. **b** Weight specific body mass loss in g day^−1^ kg^−1^. Values are given as the mean ± SD. *Arrow lines* indicate the time periods of snow and seawater exposure

To assess the effect of fasting on body mass, the weight of each hooded seal pup was measured on the day of capture and 3, 5, 12, 19, 26 and 33 days after capture. The initial body mass of the five seals at the start of their post-weaning fast varied from 50.77 to 37.50 kg. The body mass, as percentage of initial body mass (at time of capture), decreased exponentially in all animals (Fig. 2a). The average decrease of body mass can be described by Eq. 12 that was obtained using an exponential regression analysis, where BM is the body mass as percentage of initial body mass.

12 $$\text{BM} = 9 8. 30 9 {\text{e}}^{ - 0.00 9t} \quad \quad \left( {r \, = \, 0. 9 8} \right).$$

During the first 12 days of the experiment, when the animals had access to snow, the weight specific body mass loss changed from about 17 to 7 g day^−1^ kg^−1^. During the last 21 days of the experiment, the weight loss stabilized to around 9 g day^−1^ kg^−1^ (Fig. 2b).

Physiological parameters related to water balance and water flux in five hooded seals, during their post-weaning fast, were measured and calculated with the tritiated water method (Table 1). The average fractional turnover rate of tritiated water (*K*_2H_) in the five seals did not change, being 0.034 d^−1^ ± 0.009 (SD) and 0.039 d^−1^ ± 0.001 (SD) (*P* > 0.05) during snow and seawater exposure, respectively (Table 1; Fig. 1). Thus, the rate in which the isotope left the body did not significantly change upon seawater exposure. This small change coincides with the limited change in the biological half-time (*t*_1/2_) of tritiated water in the body pool. The half-time was 21.5 days ± 4.3 (SD) during snow exposure and 17.9 days ± 0.4 (SD) during seawater exposure (*P* > 0.05) (Table 1).

**Table 1:** The average physiological parameters of five hooded seal pups during the first 12 days of their 33 day post-weaning fast when all animals had ad lib access to snow

BM_start_ (kg)	BM_end_ (kg)	*N*_start_ (l)	*N*_end_ (l)	*K*_2H_ (d^−1^)	*K*_*N*_ (d^−1^)	$t_{2}^{1}$ (d)	MR (kcal day^−1^)
$$\bar{X}_{snow}$$							
43.2 ± 5.3	38.4 ± 4.8	15.7 ± 1.9	13.5 ± 1.8	0.034 ± 0.009	0.013 ± 0.007	21.5 ± 4.3	1862 ± 417
$$\bar{X}_{\text{seawater}}$$							
38.4 ± 4.8	31.3 ± 4.5	13.5 ± 1.8	11.4 ± 1.0	0.039 ± 0.001	0.008 ± 0.006	17.9 ± 0.4	1821 ± 380

The average parameters of four hooded seal pups during the last 21 days of their post-weaning fast when they had ad lib access to seawater

*BM*_*start*_ body mass at start of snow or seawater exposure period, *BM*_*end*_ body mass at end of snow or seawater exposure period, *N*_*start*_ total body water at start of snow or seawater exposure period, *N*_*end*_ total body water at end of snow or seawater exposure period, *K*_*2H*_ fractional turnover rate of tritiated water, *K*_*N*_ fractional turnover rate of body water, $t_{2}^{1}$ biological half-time of tritiated water in the body pool, *MR* daily metabolic rate based on protein and fat loss during the separate periods of the hooded seals fast

**Fig. 3:**
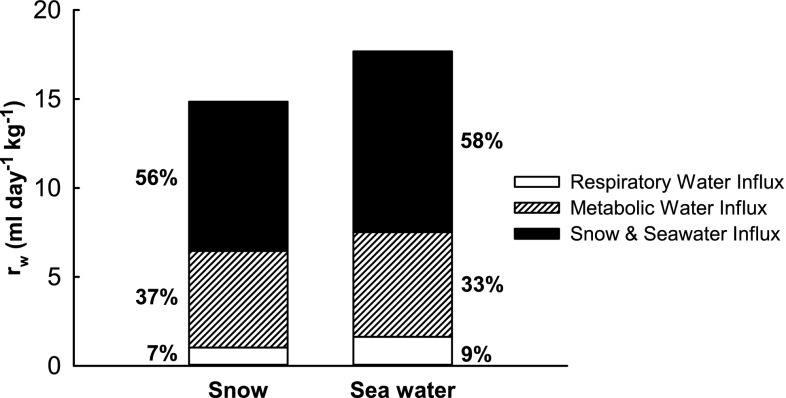
Weight specific water influx rates during snow and seawater exposure. Influx rates per route in mL day^−1^ kg^−1^. *Percentages* indicate proportional contribution of the influx route to the total influx rate of 15 mL day^−1^ kg^−1^ during snow exposure and 18 mL day^−1^ kg^−1^ during seawater exposure

Weight specific influx of water was 15 mL day^−1^ kg^−1^ during snow exposure and 18 mL day^−1^ kg^−1^ during seawater exposure (Fig. 3), amounting to around 607 mL day^−1^ during the fasting period (Table 2). Of the total water influx, approximately 56% (i.e. 8 mL day^−1^ kg^−1^) was due to the ingestion of snow and 58% (i.e. 10 mL day^−1^ kg^−1^) was due to the ingestion of seawater (Fig. 3). Thus, snow and seawater contribute to a significant proportion of daily influx.

**Table 2:** Water efflux and influx rates during 12 days of snow exposure and 21 days of seawater exposure

$r_{{{\text{H}}_{ 2} {\text{O}}}}$	*r*_w_	H_2_O_respiratory−influx_	H_2_O_metabolic_	H_2_O_snow/seawater_
Snow				
791 ± 139	604 ± 119	42 ± 7	221 ± 53	341 ± 63
Seawater				
718 ± 222	610 ± 142	56 ± 7	203 ± 48	351 ± 195

Values are given as the mean in ml day^−1^ ± standard deviation

$r_{{H_{2} O}}$ total water efflux rate, *r*_*w*_ total water influx rate, *H*_*2*_ *O*_*respiratory−influx*_ respiratory water influx rate, *H*_*2*_ *O*_*metabolic*_ metabolic water influx, *H*_*2*_ *O*_*snow/seawater*_ water influx through snow or seawater ingestion

**Fig. 4:**
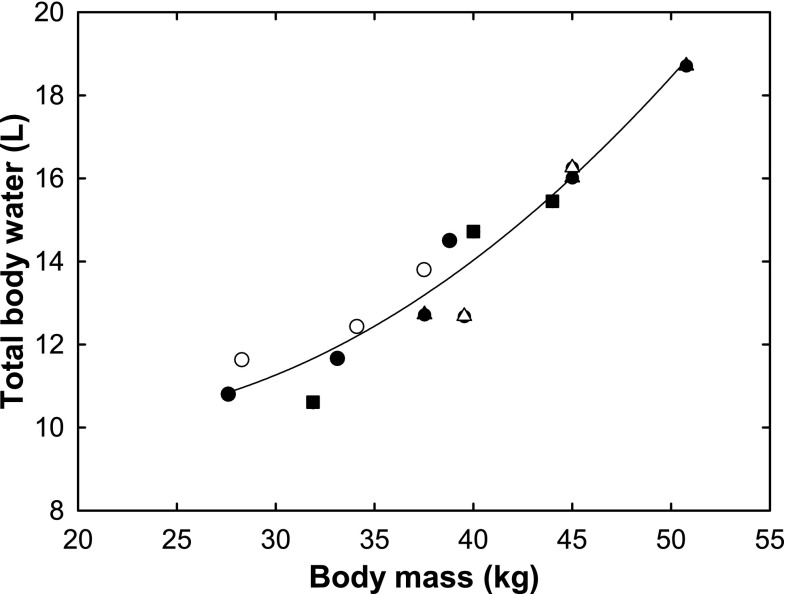
Total body water (L) in relation to body mass (kg) of five hooded seal pups during their post-weaning fast. A curvilinear regression based on the average of all five seals is described by: *N* = 0.0082BM^2^–0.2995BM + 12.845 (*R* ^2^ = 0.93) where *N* is total body water and BM is body mass

To determine changes in total body water in relation to body mass throughout the fast, total body water was plotted against body weight of all five seals. A curvilinear regression line was found (Fig. 4). This relationship indicates that leaner hooded seal pups, towards the end of their fast, consist proportionally of more protein than at the beginning of their fast when they are heavier, assuming that the free water content in protein (73%) is much higher than in fat (10%). This corresponds with the decreased protein catabolism towards the end of their fast, during seawater exposure. The hooded seal pups lost on average 1.32 g day^−1^ kg^−1^ ± 0.75 (SD) of protein during snow exposure, in the first 12 days of the experiment, compared to 0.81 g day^−1^ kg^−1^ of ± 0.83 (SD) of protein during seawater exposure. The energy derived from protein catabolism as percentage of the daily metabolic rate was 15% ± 8.1 (SD) in the beginning of their fast, and 10% ± 10.3 (SD) towards the end of their fast.

**Fig. 5:**
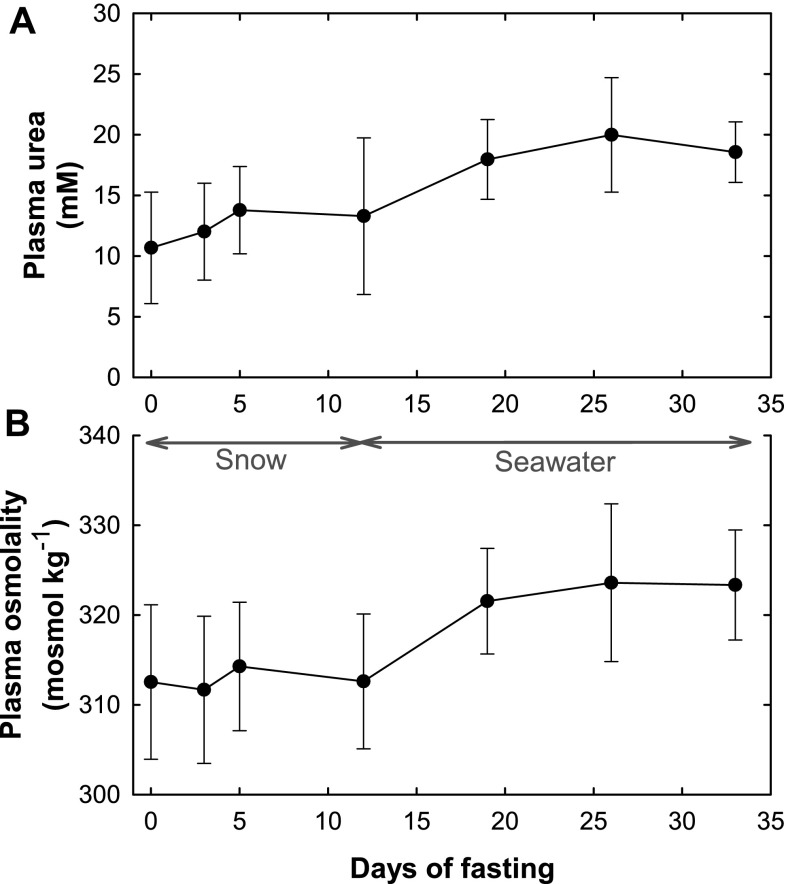
**a** Average plasma urea concentration (mM) of five hooded seal pups during their 33 day post-weaning fast ± SD. **b** Average plasma osmolality (mosmol kg^−1^) of five hooded seal pups during their 33 day post-weaning fast ± SD. *Arrow lines* indicate the time periods of snow and seawater exposure

**Fig. 6:**
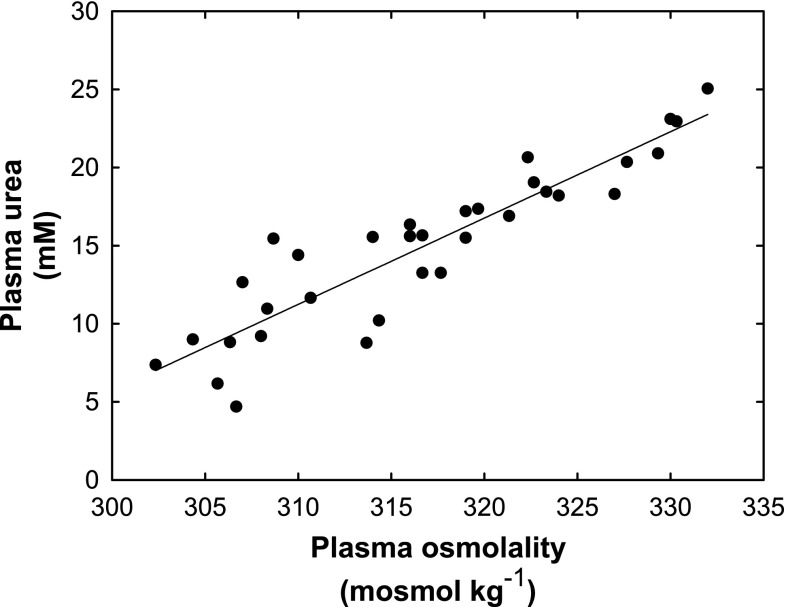
Plasma urea concentration in relation to plasma osmolality of five hooded seal pups during their post-weaning fast. A linear regression based on the average of the five seals is included, showing a significant (*P* < 0.001) correlation between the two parameters (*R* ^2^ = 0.83)

The hematocrit percentage remained stable around 60% throughout the entire study. The plasma urea concentration showed a significant increase from 10.7 mmol L^−1^ ± 4.6 (SD) to 18.6 mmol L^−1^ ± 2.5 (SD) (*P* = 0.021) from the beginning to the end of the fast (Fig. 5a). The plasma osmolality remained relatively constant around 313 mosmol kg^−1^ ± 8.60 (SD) during snow exposure but increased significantly to 323 mosmol kg^−1^ ± 6.13 (SD) (*P* = 0.003) during seawater exposure (Fig. 5b). A significant correlation was found when the plasma osmolality was related to the plasma urea (*P* < 0.001) (Fig. 6).

## Discussion

It has long been thought that marine mammals do not voluntarily drink seawater (Albrecht 1950; Irving et al. 1935). Studies performed in the second half of last century, however, showed that mariposia does occur in cetaceans, pinnipeds, and sea otters (Costa 1982; Gentry 1981; How and Nordøy 2007; Hui 1981; Renouf et al. 1990; Skalstad and Nordøy 2000; Tefler et al. 1970). Suggested reasons for mariposia in marine mammals are to facilitate thermal regulation (Gentry 1981), to maintain electrolyte balance (Ridgway 1972; Ridgway and Venn-Watson 2010; Skalstad and Nordøy 2000) and to increase urinary osmotic space for the excretion of urea (Costa 1982; Storeheier and Nordøy 2001). In addition, How and Nordøy (2007) showed that experimentally dehydrated adult harp seals were able to restore water balance with ad lib access to seawater. The aims of this study were to determine if, and to what extent, hooded seal pups ingest snow and seawater during their post-weaning fast, and to determine the effect of ingested snow and seawater on their water balance.

The tritiated water method used in this study is based on several assumptions as outlined by Lifson and McClintock (1966) and Nagy and Costa (1980). In this study, all the assumptions were considered and evaluated using the same approach as Nordøy et al. (1992) and Skalstad and Nordøy (2000). It is concluded that the potential errors mentioned in these papers will cancel each other out and will thus not affect the conclusions made in this study. In addition, total body protein and total body fat were calculated following Reilly and Fedak (1990) (Eqs. 9, 10). Assuming free water content of 73% in protein and 10% in fat (Pace and Rathbun 1945), the protein and fat wet weights were calculated. The calculated average daily protein and fat loss between day 1 and 12 of fasting, i.e. during snow exposure, was less than 5% different from the actual average daily body mass loss. Between day 12 and 33 of fasting, i.e. during seawater exposure, the calculated average daily protein and fat loss was 7% different from the actual average daily body mass loss.

The main finding in our study is that the arctic hooded seal pups eat snow and drink seawater in significant amounts during their post-weaning fast. Hooded seal pups from the Greenland Sea population spend the first few weeks of their life fasting in the pack ice, after which they become pelagic and migrate in open water to the feeding grounds (Folkow et al. 2010), most likely still fasting. The transition from the pack ice environment to a marine habitat with access to only seawater was simulated in our study design. In this study, we showed that recently weaned hooded seal pups, with access to snow obtain on average 56% of the total water influx of 15 mL day^−1^ kg^−1^ though the ingestion of this snow. Likewise, when the hooded seal pups thereafter only had access to seawater, the drinking of this water contributed to 58% of the total water influx of 18 mL day^−1^ kg^−1^.

The total daily water influx found in this study is similar to the 16.1 mL day^−1^ kg^−1^ found in the first days of the post-weaning fast of wild hooded seal pups (Lydersen et al. 1997), indicating that the animals in this study have the same turnover rate as free-living animals under the same conditions. Moreover, the average rate of body mass loss found in this study was 0.36 kg day^−1^. This value is equal to the 0.4 kg day^−1^ found in free-living hooded seal pups by Bowen et al. (1987) and Oftedal et al. (1989) during the first four to five weeks of the post-weaning fast.

It has long been assumed that marine mammals can maintain water balance from the ingestion of free water in the food and from metabolically derived water (Depocas et al. 1971; Fetcher 1939; Irving et al. 1935; Ortiz et al. 1978; Pilson 1970; Smith 1936). The main reason to assume that the intake of fresh or salt water does not occur in pinnipeds is that these species have several mechanisms to limit their water loss. They prevent dehydration through low cutaneous evaporative water loss (Matsuura and Whittow 1974). They have efficient nasal heat exchange and an apneic breathing pattern, both limiting respiratory water loss (Coulombe et al. 1965; Folkow and Blix 1987; Huntley et al. 1984; Lester and Costa 2006; Skog and Folkow 1994), and they have the ability to produce urine with an increased osmolality, which reduces obligatory urinary water loss (Bester 1975; Hong et al. 1982; Ridgway 1972). All of which result in a long biological half-time of body water (*t*_1/2_) (Nordøy et al. 1992; Ortiz et al. 1978). In this study, on the other hand, the biological half-time of body water (*t*_1/2_) was 21.5 and 17.9 days during snow and seawater exposure, respectively. These values are considerably shorter than the 53.5 days found in fasting northern elephant seal pups (Ortiz et al. 1978) and 38.2 days found in fasting grey seals (*Halichoerus gruypus*) pups (Nordøy et al. 1992). The terrestrial fasting northern elephant seal and grey seal pups in those studies did not have access to exogenous water and still manage to maintain homeostasis, this being achieved by the efficient water saving mechanisms mentioned above.

In their natural environment hooded seal pups have access to exogenous water during their post-weaning fast. Although the hooded seal pups in this study had a short *t*_1/2_, they did not become dehydrated, further supporting the ingestion of snow and seawater. The conclusion that the animals did not become dehydrated is based on the fact that there was no decrease in total body water in relation to body mass (Fig. 4) and the haematocrit values remained constant, whereas they would have increased in dehydrated animals. But most importantly, there was no profound increase in plasma urea concentration (Fig. 5a) nor in the plasma osmolality (Fig. 5b). Grey seal pups maintain stable plasma urea and osmolality values during a post-weaning fast of the same duration when kept without exogenous water (Nordøy et al. 1992). In harp seals, on the other hand, the plasma urea concentration increases from the very start of the post-weaning fast when no exogenous water is provided (Nordøy et al. 1993). These findings suggest that arctic seal species become dehydrated without access to exogenous water during their post-weaning fast.

Why do these arctic seal species need exogenous water to maintain water balance during their post-weaning fast? One possible explanation is that the obligatory urinary water loss is higher in fasting hooded seals pups than in fasting grey and elephant seals pups. In fasting marine mammals, the proportion of protein oxidation is low compared to the fat oxidation. During their post-weaning fast, grey seal pups derive 6% of their energy requirements through the oxidation of protein (Nordøy and Blix 1985; Nordøy et al. 1990). In northern elephant seal pups, this value is below 4% during the first week of their post-weaning, and lower than 2% towards the end of the fast (Adams and Costa 1993; Pernia et al. 1980). In this study, on the other hand, we found that the energy derived from protein catabolism was almost 15% during the first 12 days of the fast and 10% during the final 21 days. This suggests that the arctic hooded seal pup derives a higher proportion of energy from protein catabolism during the post-weaning fast than grey and elephant seal pups. A higher proportion of protein catabolism increases the nitrogen load on the kidneys, which necessitates the production of a larger urine volume to eliminate urea at the expense of body water. This may be one explanation for why arctic seal species ingest exogenous water, i.e. to supplement their metabolic water production. In addition, oxidation of fat provides 1.1 g of water per gram fat oxidized while the oxidation of protein yields 0.4 g water per gram protein (Kleiber 1975). The non-arctic seal species, with a higher proportion of fat oxidation, produce thus relatively more metabolic water which they are able to use to eliminate the end products of protein catabolism. Storeheier and Nordøy (2001) showed that fasting adult harp seals have an increased urea excretion after the ingestion of seawater compared to controls. They suggested that seawater might be an exogenous water source to increase the urinary osmotic space, a theory originally proposed by Wolf et al. (1959). The ingestion of seawater, as well as snow and ice, has also been reported in captive adult harp seals (Gales and Renouf 1993; Renouf et al. 1990).

A contributing explanation is that the ingestion of snow and seawater is necessary to compensate for the reduced respiratory water influx in arctic seals compared to non-arctic seals. Due to the low temperature, arctic phocids are breathing almost dry air. In temperate/sub-tropical regions, however, phocids are breathing air with an absolute humidity of 20–40 mL H_2_O per m^3^ air. In the current experimental study, the average air temperatures was around 3 °C which translates into an absolute humidity of about 5.5 mL H_2_O per m^3^ air (with 100% RH), giving a respiratory water influx of 7–9% of the total water influx (Fig. 3). In the wild, however, hooded seal pups are born in air temperatures of −20 °C. Under these temperatures, even with a relative humidity of 100%, the absolute humidity is nearly zero which means that no water enters free-living hooded seal pups through the respiration during their post-weaning fast. In addition to the respiratory water influx, the respiratory water efflux also differs between the sub-tropical northern elephant seal pups, the temperate grey seal pups and arctic seal pups. Lester and Costa (2006) estimated that fasting elephant seal pups loose on average 50 g of water per day through respiratory evaporation, which equals a weight specific respiratory water efflux of about 0.5 mL day^−1^ kg^−1^. Using Eqs. 6 and 7, the weight specific respiratory water efflux of the seals in this study (ambient temperature ranging between 0 and 6 °C) can be estimated to 6 mL day^−1^ kg^−1^. In the wild, however, an ambient temperature of −10–20 °C is more likely for arctic breading species. Using the data from Folkow and Blix (1987, 1989) it can be derived that grey and harp seals lose about 2 mL day^−1^ kg^−1^ through respiration under such ambient temperatures, still being four times as high as in the fasting northern elephant seals. This further supports the conclusion that arctic seals ingest snow and seawater, i.e. to compensate for the reduced respiratory water influx and relatively high respiratory water efflux.

We conclude that hooded seal pups depend on snow and seawater for maintenance of water balance during the first few weeks of life when they undergo their post-weaning fast. This arctic seal species is born in an environment surrounded by fresh and marine water where the ingestion of snow and seawater may be necessary for urinary water production, to aid in the excretion of urea, and to compensate for the relatively high net loss of respiratory water.

## Electronic supplementary material

Below is the link to the electronic supplementary material.

Supplementary material 1 (MOV 59270 kb)

## Abbreviations

RH: Relative humidity
S.A.: Specific activity
TBF: Total body fat
TBP: Total body protein
